# Multipotency of mouse trophoblast stem cells

**DOI:** 10.1186/s13287-020-1567-4

**Published:** 2020-02-13

**Authors:** Minmin Hou, Junwen Han, Gu Li, Min-Young Kwon, Jiani Jiang, Sirisha Emani, Elizabeth S. Taglauer, Jin-Ah Park, Eun-Bee Choi, Munender Vodnala, Yick W. Fong, Sitaram M. Emani, Ivan O. Rosas, Mark A. Perrella, Xiaoli Liu

**Affiliations:** 1 Department of Medicine, Division of Pulmonary and Critical Care Medicine, Brigham and Women’s Hospital, 75 Francis Street, Boston, MA 02115 USA; 2 Department of Obstetrics and Gynecology, West China Second University Hospital, Sichuan University, Chengdu, China; 3 Department of Cardiovascular Surgery, Children’s Hospital, Boston, MA USA; 4 Division of Newborn Medicine, Boston Children’s Hospital, Boston, MA USA; 5 Department of Environmental Health, Harvard School of Public Health, Boston, MA USA; 6 Department of Medicine, Division of Cardiovascular Medicine, Brigham and Women’s Hospital, Boston, MA USA; 7 Harvard Stem Cell Institute, Cambridge, MA USA; 8 Department of Pediatric Newborn Medicine, Brigham and Women’s Hospital, Boston, MA USA

**Keywords:** Stem cells, Trophoblast cells, Multipotency, In utero

## Abstract

**Background:**

In a number of disease processes, the body is unable to repair injured tissue, promoting the need to develop strategies for tissue repair and regeneration, including the use of cellular therapeutics. Trophoblast stem cells (TSCs) are considered putative stem cells as they differentiate into other subtypes of trophoblast cells. To identify cells for future therapeutic strategies, we investigated whether TSCs have properties of stem/progenitor cells including self-renewal and the capacity to differentiate into parenchymal cells of fetal organs, in vitro and in vivo.

**Methods:**

TSCs were isolated using anti-CD117 micro-beads, from embryonic day 18.5 placentas. In vitro, CD117^+^ TSCs were cultured, at a limiting dilution in growth medium for the development of multicellular clones and in specialized medium for differentiation into lung epithelial cells, cardiomyocytes, and retinal photoreceptor cells. CD117^+^ TSCs were also injected in utero into lung, heart, and the sub-retinal space of embryonic day 13.5 fetuses, and the organs were harvested for histological assessment after a natural delivery.

**Results:**

We first identified CD117^+^ cells within the labyrinth zone and chorionic basal plate of murine placentas in late pregnancy, embryonic day 18.5. CD117^+^ TSCs formed multicellular clones that remained positive for CD117 in vitro, consistent with self-renewal properties. The clonal cells demonstrated multipotency, capable of differentiating into lung epithelial cells (endoderm), cardiomyocytes (mesoderm), and retinal photoreceptor cells (ectoderm). Finally, injection of CD117^+^ TSCs in utero into lungs, hearts, and the sub-retinal spaces of fetuses resulted in their engraftment on day 1 after birth, and the CD117^+^ TSCs differentiated into lung alveolar epithelial cells, heart cardiomyocytes, and retina photoreceptor cells, corresponding with the organs in which they were injected.

**Conclusions:**

Our findings demonstrate that CD117^+^ TSCs have the properties of stem cells including clonogenicity, self-renewal, and multipotency. In utero administration of CD117^+^ TSCs engraft and differentiate into resident cells of the lung, heart, and retina during mouse development.

**Electronic supplementary material:**

The online version of this article (10.1186/s13287-020-1567-4) contains supplementary material, which is available to authorized users.

## Background

There is a great need to promote tissue repair and regeneration after tissue damage, with particular challenges in patients in which sources of autologous cells are not feasible. A variety of stem cells including embryonic stem cells (ESCs), induced pluripotent stem cells (iPSCs), and adult stem cells have been studied for their treatment potentials in many different diseases [1, 2]. Placental stem cells have attracted a great deal of interest by scientists in the past decade [3–5]. The placenta is a discarded organ after birth and does not carry the same ethical concerns as other embryonic tissue. Mouse or human placenta-derived cells have been reported to express very low or non-existent levels of the classical major histocompatibility complex (MHC), implicating immune privilege [6–8]. It may be a useful cell for allogeneic transplantation, with broad prospects for therapeutic application as seen with mesenchymal stem/stromal cells. This is especially important as not all stem cells can be used allogeneically, and the tissue of injury may not be a viable source of stem cells. In certain disease processes, autologous stem cells, even if accessible, may not be optimal for use due to an underlying genetic disorder. Moreover, placenta-derived cells have less risk for tumorigenesis than that of embryonic stem cells [9–11]. Compared to adult/organ-specific stem cells, placenta-derived stem cells appear to have better proliferation and differentiation potential, as well as potential paracrine actions [12, 13].

A variety of stem/progenitor cells have been discovered in mouse and human placentas, including trophoblast stem cells (TSCs), endothelial stem cells, amniotic epithelial cells (AECs), and perivascular stem cells [9, 12–16]. Recent investigations have focused on placenta-derived mesenchymal stromal cells (MSCs) from different regions of the placenta, AECs, and hematopoietic stem cells (HSCs) of the umbilical cord blood [16–18]. Although umbilical cord blood-derived HSCs are mainly used for blood system diseases, non-hematological diseases have also been studied. It is estimated that approximately 115,000 subjects have received UCB-derived HSCs in 2012 [19], for applications ranging from immunomodulation to tissue regeneration [16, 20–22]. In addition, MSCs derived from umbilical cord, amniotic fluid, and Wharton’s jelly have been undergoing clinical trials for the regeneration of nerves, hepatocytes, and cardiomyocytes [22–25], and most prominently for the immune regulation of systemic inflammatory diseases [26, 27]. Both laboratory studies and clinical trials have shown encouraging results comparable to the standard bone marrow-derived MSCs [28]. While MSCs are considered to have an important role in the modulation of immune function and to be protective of tissue injury, their ability to engraft and replace damaged tissue in vivo is very limited [12, 29].

For the application of cellular therapeutics in congenital disorders with devastating in utero consequences, both engraftment and paracrine actions of the transplanted cells may well be required for benefit. CD117, a surface marker of the stem/progenitor cells [30, 31], has been found in cells of mouse and human placentas [12, 18, 32–35]. It is believed that these cells are CD34 positive, lineage negative, and function as HSCs, important for the circulatory system in the early development stage. Recently, a subpopulation of trophoblast cells (TCs) expressing stem cell antigen-1 (Sca1, a stem cell marker) in the mid-gestation placenta was identified, and these cells are capable of differentiating into other trophoblast placental subtypes [35]. In the present study, we investigated a population of TCs expressing CD117, allowing isolation of a placental cell with stem cell properties, including self-renewal, clonogenesis, and multipotency in vitro and in utero after cell transplantation into fetal organs.

## Materials and methods

### Immunocytochemistry

Immunocytochemistry was performed as previously described [36–38]. Cells were fixed with 4% paraformaldehyde at room temperature (RT) for 10 min, and for intracellular markers, permeabilization of cells was carried out with 0.2% Triton X-100 at RT for 10 min. Next, cells were blocked in 10% donkey serum at RT for 30 min and then incubated in the primary antibodies at RT for 1 h or at 4 °C overnight. Primary antibodies were applied in this study: CD117 (R&D Systems, Cat. AF1356, 1:50), caudal type homeobox 2 (CDX2; Abcam, Cat. Ab76541, 1:200), aquaporin 5 (AQP5; EMD Millipore, Cat. AB15858, 1:100), prosurfactant protein C (SPC; Abcam, Cat. ab40879, 1:100), tight junction protein-1 (ZO-1; Thermo Fisher Scientific, Cat. 40-2200, 1:400), cardiac troponin T (cTnT; Abcam, Cat. ab125266, 1: 200), sarcomeric α-actinin (Sar α-actinin; Sigma Cat. A7811, 1:200), rhodopsin (Rho; Abcam, Cat. ab3267, 1: 200), recoverin (Rec; EMD Millipore, Cat. AB5585, 1: 500), arrestin 1 (Arr1; Abcam, Cat. Ab32099, 1:200). Second antibodies were conjugated with fluorescence, then were incubated with samples at 37 °C for 1 h. Nuclei were stained with 4′,6-diamidino-2-phenylindole (DAPI; 1:1000 in PBS) at 37 °C for 10 min. Images were analyzed using fluorescence microscopy or a confocal microscopy system (Olympus).

### Immunohistochemistry

Immunohistochemistry was performed as previously described [36, 37, 39]. Placentas from pregnant mice at E18.5 were harvested, formalin fixed, dehydrated, and embedded in paraffin. Tissue sections, 5 μm in thickness, were cut and deposited on poly-lysine-coated slides. To improve antigen recognition, the sections were microwaved in 10 mM citrate buffer (pH 6.0) for 10~12 min. They were blocked with 10% donkey serum and then incubated with primary antibodies at 4 °C overnight. Primary antibodies were applied in this study: CD117 (R&D Systems, Cat. AF1356, 1:50), cytokeratin 7 (CK7; Abcam, Cat. ab181598, 1:1000), Ki67 (Dako, Cat. M7249, 1:100), CD34 (Abcam, Cat. ab8158, 1:50), CD45 (Abcam, Cat. ab10558, 1:50), CD31 (Abcam, Cat. ab28364, 1:50), vimentin (Abcam, Cat. ab92547, 1:200), E-cadherin (Abcam, Cat. ab53033, 1:50), cardiac troponin I (cTnI, Abcam, Cat. ab47003, 1:200), SPC, and Rho (see “Immunocytochemistry”). Finally, species-matched secondary antibodies conjugated with fluorescence were applied at 37 °C for 1 h. Nuclei were stained with DAPI. A 3,3′-diaminobenzidine system was also carried out for CD117 staining. Images were analyzed using a fluorescence or light microscopy or a confocal microscopy system (Olympus).

### Isolation and culture of CD117^+^ TSCs in vitro

Cell culture was performed as previously described with modifications [37, 40, 41]. Placentas were harvested from pregnancies of mice at E18.5. Placentas were chopped into small pieces after trimming off ~ 1 mm of the maternal portion and dissociated in an enzyme buffer containing collagenase I (1 mg/ml) and collagenase II (2.5 mg/ml, Worthington Biochemical) in PBS. The cells were expanded in DMEM/F12 (Lonza) supplemented with 20% fetal bovine serum (FBS; GE Healthcare), 100 mM nonessential amino acids (Cellgro), 55 mM beta-mercaptoethanol (Gibco), 1 mM sodium pyruvate (Cellgro), 10 ng/ml LIF (Millipore), 20 ng/ml murine basic fibroblast growth factor (bFGF; PeproTech), and 1% penicillin/streptomycin/l-glutamine (Corning). Culture medium was changed every other day. CD117^+^ TSCs were isolated using anti-mouse CD117 MicroBeads (Miltenyi Biotec, Cat. 130-091-224, 20 μl per 10^7^ cells) [37].

### Flow cytometry

Cells were detached from culture dish using HyQtase and blocked with Fc (CD32/16) at RT for 15 min, followed by incubating with primary antibodies conjugated with fluorescence at 4 °C for 30 min in darkness [37]. The cells were then assessed using BD FACS Canto II, and the data was analyzed using FlowJo software (TreeStar). Analyses included CD117 (Abcam, Cat. ab5506, 1:10), Sca1 (BioLegend, Cat. 108111, 1:50), CD73 (BioLegend, Cat. 127209, 1:50), CD90.2 (BioLegend, Cat. 105307, 1:50), CD105 (BioLegend, Cat. 120407, 1:50), CD34 (BioLegend, Cat. 128611, 1:50), CD31 (BioLegend, Cat. 102407, 1:50), CD45 (BioLegend, Cat. 103111, 1:50), CD11b (BioLegend, Cat. 101207, 1:50), MHC I (eBioscience, Cat. 17-5957-80, 1:50), and MHC II (eBioscience, Cat. 17-5957-82, 1:50).

### Growth analysis

For the studies assessing cell growth, CD117^+^ TSCs were plated on dishes (15,000 cells per 35-mm^2^ dish, triplicate for each time point), and the cells were placed in growth medium and then counted daily for 5 days.

### Limiting dilution and clone formation in vitro

Limiting dilution for clone formation was performed as we previously described [37]. Briefly, 100 CD117^+^ TSCs were plated in 100-mm-diameter dish (the density, ≈1 cell/60 mm^2^) to obtain multicellular clones derived from a single founder cell. Growth medium was changed every 3 days and the clones formed over 2–3 weeks (see isolation and in vitro culture of CD117^+^ TSCs), and then imaged by phase-contrast microscopy and fluorescent microscopy for CD117 immunostaining (see “Immunocytochemistry”). Clonal cells were also isolated using cloning cylinders (Sigma) and expensed in growth culture medium.

### Crystal violet staining for clones

The dish with clones was placed on ice and washed twice with cold PBS, and then fixed with pre-chilled 100% methanol at − 20 °C for 10 min. After removing the methanol, the dish was incubated with 0.5% crystal violet solution in 25% methanol at RT for 10 min and rinsed with water until the unbound blue stain was gone. Colonies with more than 50 cells per clone were counted.

### Differentiation of CD117^+^ TSCs in vitro

#### Airway epithelial cell differentiation

Airway epithelial cell differentiation assay was performed as previously described [38]. CD117^+^ TSCs were seeded on the apical side of a clear 12-transwell plate (ThermoFisher) at a density of 65,000 cells/well and cultured in growth medium for 2 days. Then cells were exposed to 2% FBS DMEM/F12 differentiation medium supplied with 5 μg/ml insulin (Sigma), 10 μg/ml transferrin (Sigma), 30 nM sodium selenite (Sigma), 10 nM hydrocortisone (Sigma), 10 nM beta-estradiol (Sigma), 10 nM HEPES (FisherBiotech), 2 mM l-glutamine, and 50 ng/ml epidermal growth factor (PeproTech) for another 5~6 days. Once the cells reached complete confluence, the air-liquid interface was established by adding the differentiation medium to basal side and allowing the apical side to be exposed to air, for an additional 11~12 days. Cells were harvested for immunofluorescence staining (see “Immunocytochemistry”), flow cytometry, and real-time polymerase chain reaction (qRT-PCR).

#### Cardiomyocyte differentiation

Cardiomyocyte differentiation assay was performed as previously described [37]. CD117^+^ TSCs were cultured in 5% FBS DMEM/F12 containing 0.5 ng/ml bone morphogenetic protein 4 (PeproTech) for 24 h, and then exposed to 5% FBS DMEM/F12 containing 10 ng/ml bone morphogenetic protein 4, 5 ng/ml bFGF (PeproTech), and 3 ng/ml activin A (PeproTech) for an additional 4 days, followed by exposing to medium containing 10 ng/ml vascular endothelial growth factor (PeproTech) and 150 ng/ml DKK-1 (PeproTech) for another 3 days, and finally exposed to 10 ng/ml vascular endothelial growth factor, 150 ng/ml DKK-1, and 5 ng/ml bFGF for additional 2 days. Cells were harvested at day 12 for assessments of immunofluorescence staining (see “Immunocytochemistry”), flow cytometry, and qRT-PCR.

#### Cardiomyocyte differentiation in the conditioned medium of neonatal heart cells

Cardiomyocyte differentiation assay was performed as previously described [42]. Briefly, cells were harvested from day 1 neonatal hearts and cultured in Iscoves DMEM (Corning, # 10–016-CV) containing 10% FBS, 25 mM HEPES, and 1× penicillin/streptomysin/l-glutamine. Conditioned medium was collected from the cardiac cells every 24 h. CD117^+^ TSCs were then plated on a laminin-coated four-well glass slide and cultured in the conditioned medium, which was changed every other day for 3 weeks. Cells were immunofluorescence stained for cTnT and Sar a-actinin. Confocal images were taken for the assessment of striations.

#### Neural differentiation

Neural differentiation assay was performed as previously described [36] with modifications. CD117^+^ TSCs were cultured in differentiation conditions using DMEM/F12 medium supplemented with 10 ng/ml bFGF and B27 (1:50) for the next 2 days. To promote photoreceptor differentiation, cells were cultured in the differentiation medium plus N-2 supplement (1:100), 50 nM docosahexaenoic acid (Sigma), 2 μM retinoic acid (Sigma), 10 μM γ-secretase inhibitor (Sigma) for 2 days, and then changed to medium containing DMEM/F12 with B27 (1:50), 10 ng/ml nerve growth factor, 10 ng/ml insulin-like growth factor 1, and 10 ng/ml brain-derived neurotrophic factor (Sigma) for another 4–6 days. Cells were harvested for assessments of immunofluorescence staining (see “Immunocytochemistry”), flow cytometry, and qRT-PCR.

### Spheroid formation assay

Spheroid formation assay was performed as previously described [43]. Briefly, clonal CD117^+^ TSCs were resuspended in growth medium in the absence of LIF and FGFβ at a concentration of 1.25 × 10^6^ cells/ml. The drops (20 μl per drop) were hung on the cover of a culture dish for 2 days to develop the spheroids. Spheroids were then collected and plated on ultra-low attachment six-well plates and allowed to grow until day 14. Spheroids were then harvested for extraction of total RNA to perform qRT-PCR for identification of markers for germ layer differentiation.

### Real-time polymerase chain reaction

Total RNA was extracted from TSCs either before or after differentiation into lung alveolar epithelial cells, heart cardiomyocytes, or retina photoreceptor cells, using Trizol reagent (Invitrogen). cDNA was made by reverse transcription using the PuperScript III First-Strand Synthesis System (Invitrogen). qRT-PCR analyses were performed using the SYBR Green Supermix kit (Bio-Rad), and mouse gene-specific primers: CDX2: 5′-AGGCTGAGCCATGAGGAGTA-3′, 5′-CGAGGTCCATAATTCCACTCA-3′; EOMES: 5′-GGCCTACCAAAACACGGATA-3′, 5′-GACCTCCAGGGACAATCTGA-3′; ESRRB: 5′-TACCTGAACCTGCCGATTTC-3′, 5′-CCCAGTTGATGAGGAACACA-3′; CER1: 5′-TGG AACCTCCAGTAAGCCAG-3′, 5′-GGTTCATGTTCAGAAAGGGC-3′; GATA6: 5′-TTGCTCCGGTAACAGCAGTG-3′, 5′-GTGGTCGCTTGTGTAGAAGGA-3′; TBXT: 5′-CTCTAATGTCCTCCCTTGTTGCC-3′, 5′-TGCAGATTGTCTTTGGCTACTTTG-3′; BMP7: 5′-GTGGTATCGAGGGTGGAAGA-3′, 5′-ACAAGGCCGTCTTCAGTACC-3′; FGF5: 5′-TTGCGACCCAGGAGCTTAAT-3′, 5′-CTACGCCTCTTTATTGCAGCAT-3′; OTX2: 5′-CTTCATGAGGGAAGAGGTGG-3′, 5′-CTGACCTCCATTCTGCTGCT-3′; AQP5: 5′-CTCCCCAGCCTTATCCATTG-3′, 5′-CCAGAAGACCCAGTGAGAGG-3′; SPC: 5′-CAAACGCCTTCTCATCGTGGTTGT-3′, 5′-TTTCTGAGTTTCCGGTGCTCCGAT-3′; cTnT: 5′-CCTCAAGACCTGTGTGCAGT-3′, 5′-CCTCTTGCTCTTCCTGTTCC-3′; Sar α-actinin: 5′-GCTCGCCAACATGCTAATGA-3′, 5′-GCTTCAGCTGGTTCATCTGG-3′; Rho: 5′-CATGCCAATATGCCCACCTT-3′, 5′-GCACTGTGTTTCTGAACTCTTCAGA-3′; Rec: 5′-GCAGCTTCGATGCCAACAG-3′, 5′-TCATGTGCAGAGCAATCACGTA-3′.

Arrestin 1: 5′-TGAGTGTCCTCACCCAACTC-3′, 5′-AGTCAGGAAACGTGAGCAGA-3′; Arrestin 4: 5′-TGATTCTGTGCAGCTGGTTG-3′, 5′-AAGCTTCGAATGGTCTGTGC-3′.

Data were analyzed by the ΔCт threshold cycle method.

### Injections of CD117^+^ TSCs in utero

Injections of CD117^+^ TSCs were performed using the Vevo 2100 high-resolution micro-ultrasound system and a 50-MHz probe (VisualSonics, and the Vevo Integrated Rail System II with Injection System), as previously described [37]. Timed pregnancies of C57BL/6 black mice were performed to allow injections at E13.5. CD117^+^ TSCs were labeled with the green fluorescent dye PKH67 (SIGMA), which incorporates into the membrane of cells and is equally distributed to daughter cells when they divide. For the injection procedure, the dam was anesthetized, a laparotomy was performed, the uterine horn was exposed in a sterile manner, and injections were performed directly through the uterus into the lung, the walls of the heart (left ventricle, right ventricle and septum), or the sub-retinal space of eye, using a beveled glass micropipette, with a total 15,000 cells in a volume of 0.420 μl PBS. The uterine horn was then placed back into the abdomen and a normal delivery of the pups occurred 6 days later. The Harvard Medical Area Standing Committee on Animals, Harvard Medical School, approved animal care and use for all experiments in this study.

In the group of newborn pups that received injections of green fluorescently labeled TSCs at E13.5, their lungs, hearts, and eyes were harvested on day 1 after birth for immunofluorescence staining (see “Immunohistochemistry”).

### Statistical analysis

For comparisons between groups in Figs. 4b, 5b, 6b, and 7 unpaired Student’s *t* test was performed between undifferentiation and differentiation groups. Statistical significance for comparisons was accepted at *P* < 0.05. The numbers of samples per group are specified in the figure legends.

## Results

### Expression of CD117 in trophoblast cells of the E18.5 placenta

**Fig. 1:**
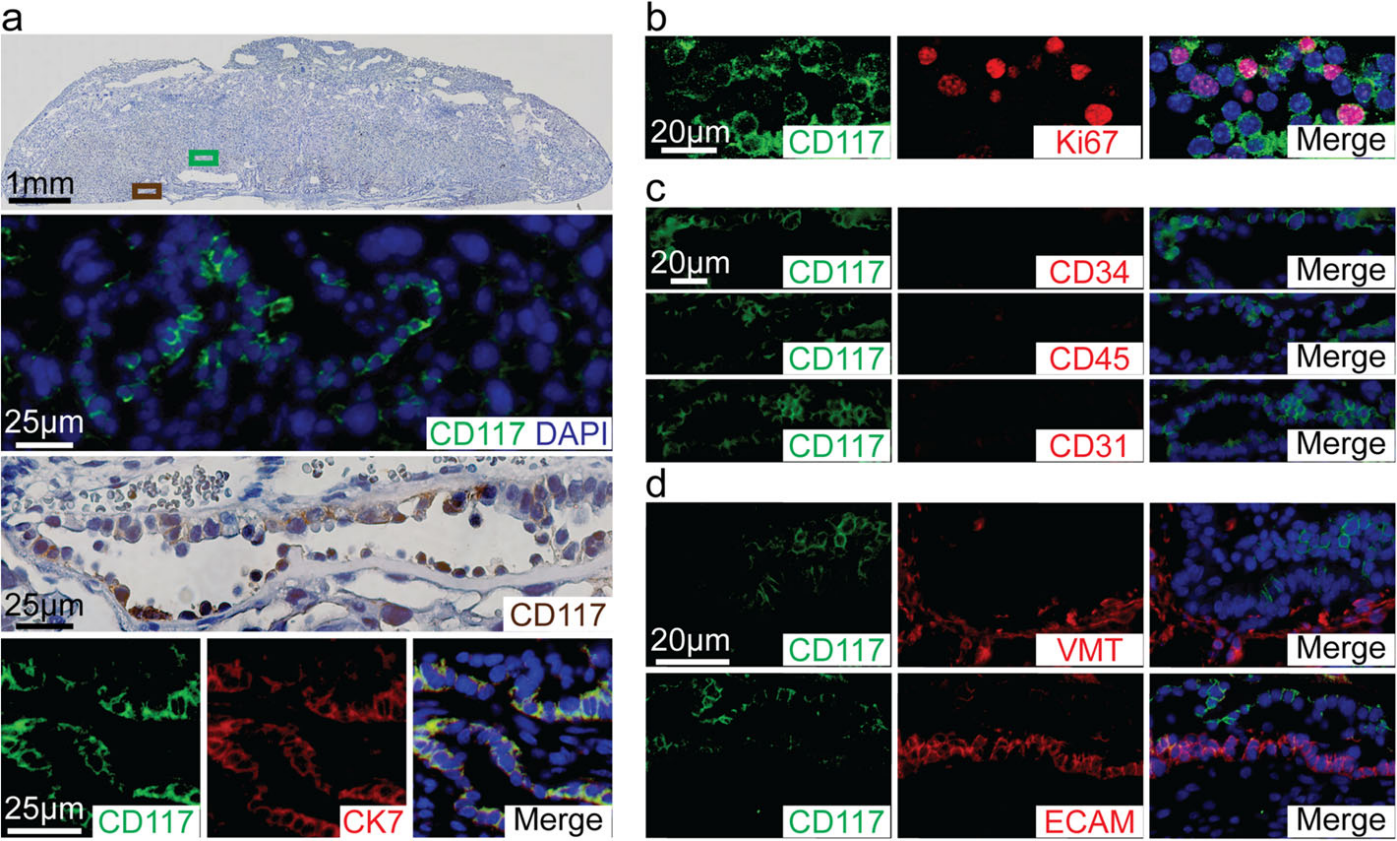
Expression of CD117 in trophoblast cells in labyrinth zone and basal chorionic plate of placenta. **a** Representative image of nuclear staining for hematoxylin, showing anatomical structure of an embryonic (E) day 18.5 placenta (top panel). A representative image of immunofluorescence staining for CD117 (green) and DAPI (nuclei, blue) from labyrinth region (second panel) depicted in the green box of the top panel. A representative image of immunohistochemical staining for CD117 (brown) and hematoxylin for nuclei (third panel), from the chorion depicted in the brown box in the top image. Bottom panels are representative images of immunofluorescence staining for CD117 (green, left) and CK7 (red, middle) and merged image with for nuclei. **b** Representative image of immunofluorescence co-staining for CD117 (green, left) with Ki67 (red, middle), merged image with DAPI (right). **c** Representative immunofluorescence image of co-staining for CD117 (green, left column) and markers for hematopoietic cells, CD34, CD45, and CD31 (red, middle column, respectively), and merged with DAPI (right column). **d** Representative immunofluorescence image of co-staining of CD117 (green, left column) and markers for MSCs, vimentin, and E-cadherin (VMT and ECAM, respectively, red, middle column) and merged with DAPI (right column)

To characterize TSCs, we performed immunohistochemical staining on the E18.5 mouse placentas. The CD117 expressing cells were found surrounding the vascular sinusoids of the labyrinth zone (Fig. 1a, green box in top panel, higher magnification in second panel), as well as within the basal chorionic plate (Fig. 1a, brown box in the top panel, higher magnification in the third panel). To confirm the TCs expressed CD117, we performed immunofluorescence co-staining with CD117 and CK7, an established TC lineage marker. CD117^+^ cells were also positive for CK7 (Fig. 1a, bottom row), and the majority of TCs in the labyrinth zone and the basal chorionic plate expressed CD117. Interestingly, immunofluorescence staining for Ki67, a cell proliferation marker, demonstrated that the majority of Ki67^+^ dividing cells in E18.5 mouse placentas were also localized in the labyrinth zone and chorionic plate of the placenta (Additional file 1: Figure S1). Next, to investigate the proliferative potential of CD117^+^ TSCs, we performed co-immunofluorescence staining for Ki67 and CD117. We found that a considerable number of CD117^+^ TSCs were positive for Ki67^+^ and that most of the Ki67^+^ cells were co-expressing CD117 (Fig. 1b). Further staining revealed the CD117^+^ cells were negative for hematopoietic and endothelial lineage markers CD34, CD45, and CD31 (Fig. 1c) and negative for mesenchymal stromal lineage markers vimentin and E-cadherin (Fig. 1d). With this repertoire of surface markers, we thus identified a CD117^+^ cell population of trophoblast lineage which demonstrates proliferative potential in situ*.*

### Characterization of CD117^+^ TSCs in vitro

**Fig. 2:**
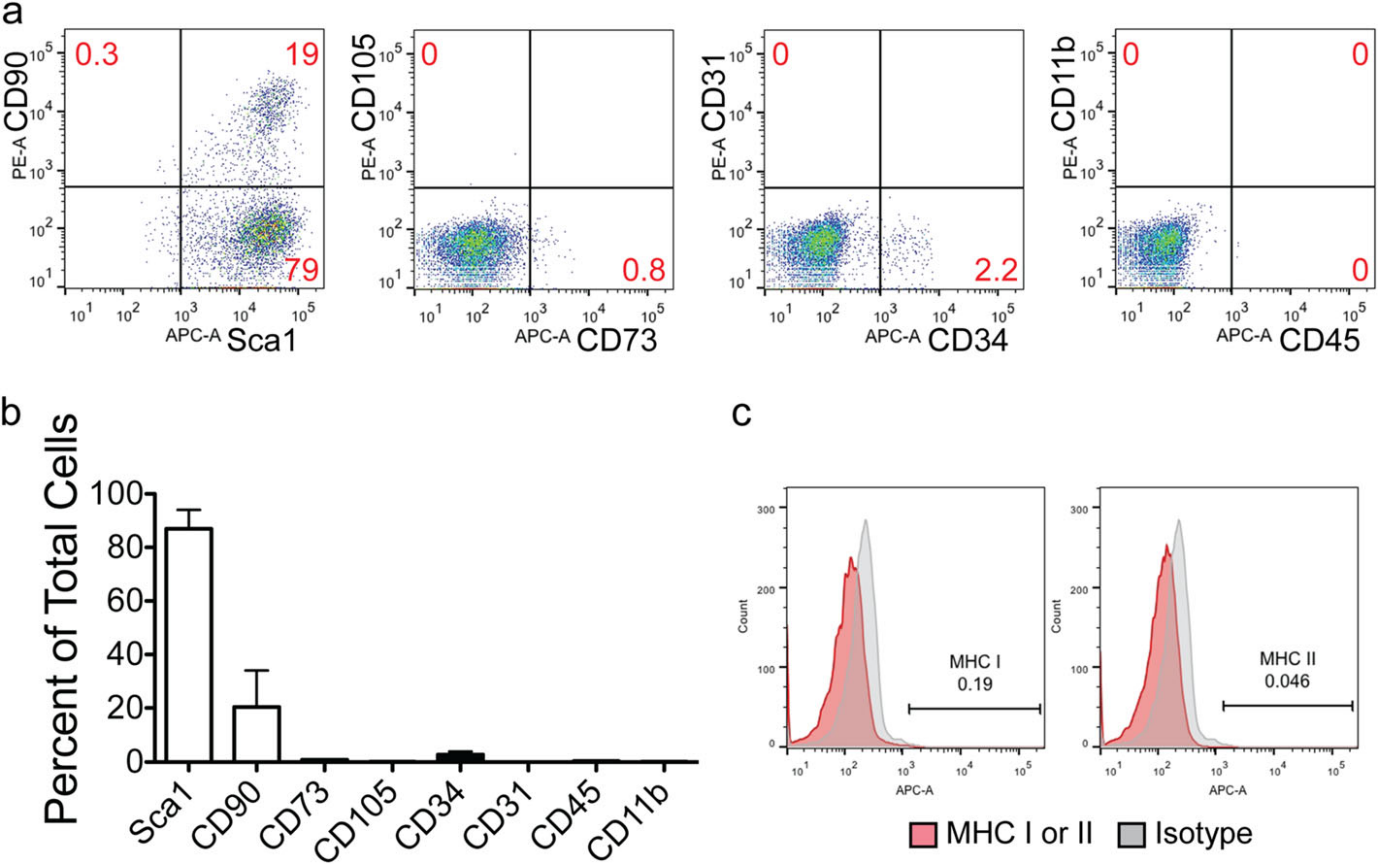
Characterization of CD117^+^ TSCs in vitro. Clonal CD117^+^ TSCs were harvested for flow cytometric analysis. **a** Representative scatter plots of flow cytometer for CD90 versus Sca1 (first panel), CD105 versus CD73 (second panel), CD31 versus CD34 (third panel), and CD11b versus CD45 (forth panel). **b** Quantitation of the flow cytometric assay showing percentage of markers for MSCs (open bars), Sca1, CD90.2, CD 105, and CD73 and hematopoietic cells (black bars), CD34, CD31, CD11b, and CD45, in total placental cells, *n* = 3~4 for each marker. **c** Representative histogram of flow cytometric assays for major histocompatibility complex classic I and II (MHC I and MHC II, respectively), gray for APC isotope control, red for MHC I and II staining (left and right, respectively)

Single-cell suspensions were collected by enzymatically dissociated of E18.5 mouse placentas and then cultured in growth medium. CD117^+^ cells were isolated using anti-CD117 magnet micro-beads, and 14.5 ± 5.8% of the cells (predominantly from the fetal portion of the placenta) were CD117^+^. We performed a growth curve of the CD117^+^ cells in culture and found that over a 5-day period there was an 18 ± 4-fold increase in the cell number (Additional file 2: Figure S2). To assess clone formation, the cells were cultured at a limited dilution. The clones were then picked and cultured in growth medium. To characterize clonal CD117^+^ TSCs in vitro*,* we performed flow cytometry (Fig. 2a), which showed that a high percentage of CD117^+^ TSCs also express Sca1 (Fig. 2b, 86.97 ± 12.23%, *n* = 4), a marker for stem cells in mice. Moreover, we performed immunofluorescence staining on CD117^+^ isolated cells and confirmed the majority of these cells express both CD117 and Sca1 (Additional file 3: Figure S3). Interestingly, TCs isolated from E18.5 placentas using a Percoll gradient, and stained with Sca1, revealed that nearly all of the TCs were positive for Sca1; however, only a subpopulation of these cells expressed CD117 (Additional file 4: Figure S4). Mathews and colleagues reported that a small portion of fetal MSCs, harvested from the chorionic villi of human placentas in the third trimester, expressed CD117 (~ 5%) [41]. Our data showed only a small subpopulation of CD117^+^ TSCs stained positive for CD90.2 and rarely expressed CD105 or CD73 (Fig. 2a, first and second panels, and b). Thus, the CD117^+^ TSCs did not fulfill the criteria of MSCs. Rare cells expressed the hematopoietic progenitor epitope CD34, the endothelial marker CD31 (Fig. 2a, third panel, and b), or the immune cell markers CD45 and CD11b (Fig. 2a, last panel, and b). These data show CD117^+^ TSCs are not of hematopoietic or vascular origin. Importantly, flow cytometry analysis demonstrated an absence of the MHC I and II in these cells (Fig. 2c).

### Self-renewal of CD117^+^ TSCs in vitro

**Fig. 3:**
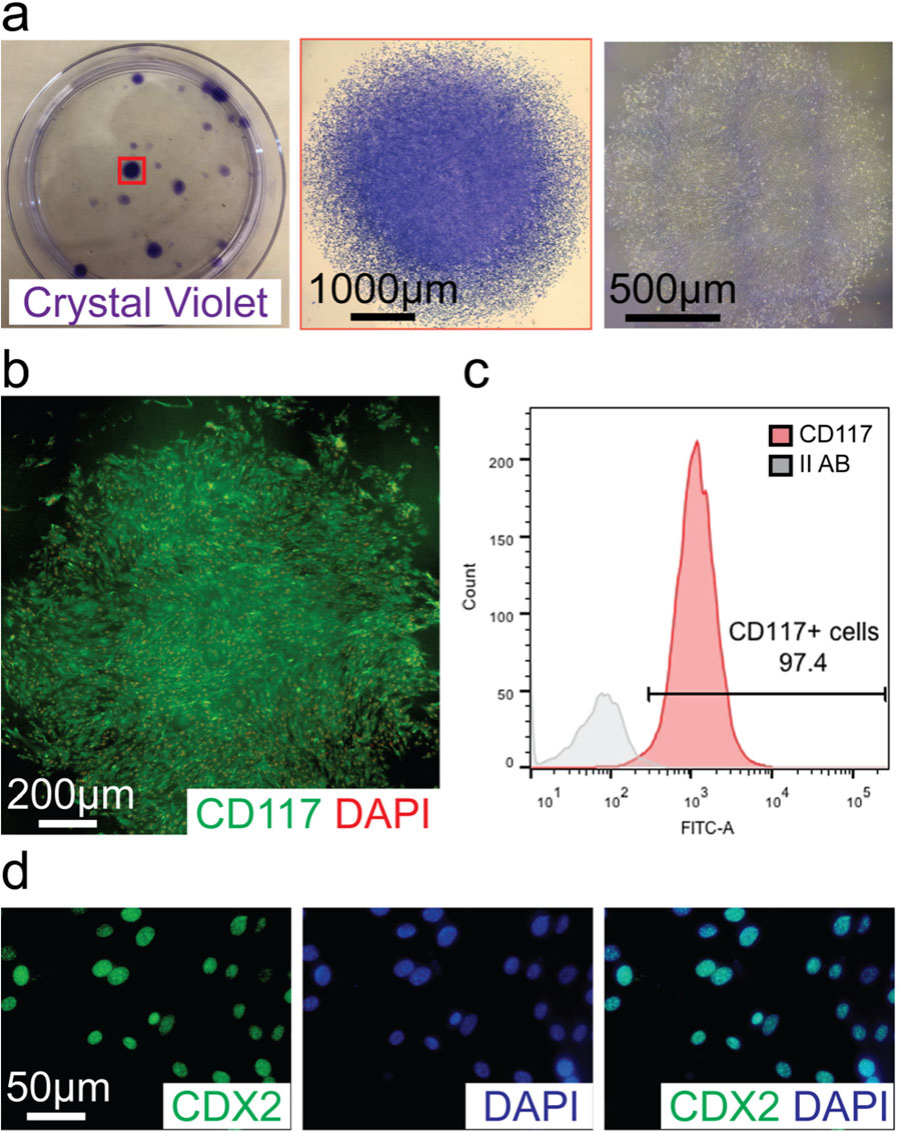
Self-renewal of placental CD117^+^ TSCs in vitro. CD117^+^ TSCs were plated in a 100-mm dish at limited dilution of one cell every 60 mm^2^ for 14 days. **a** Representative image of multicellular clones stained for crystal violet (left, purple), higher magnification of a clone staining for crystal violet (middle, purple), and phase-contrast image of an entire clone (right). **b** Representative image of a clone staining positive for CD117 (green), DAPI for nuclear staining. **c** After expansion, 97% of clonal cells are positive for CD117 as represented in flow cytometry histogram, gray for Alex 488 isotope control, red for CD117 Alex 488 staining. **d** Representative image of clonal cells staining positive for caudal type homeobox 2 (CDX2; green, left), DAPI for nuclear staining (blue, middle), and merged image (right)

Self-renewal is an important characteristic of stem cells. CD117^+^ TSCs were plated in limited dilution (density of one cell every 60 mm^2^), and over 10~14 days, multicellular clones developed, as observed by crystal violet staining (Fig. 3a, first and second panels) and light microscopy (Fig. 3a, right panel), with a clone formation efficiency of 21.33 ± 3.09% (*n* = 3) in vitro. Immunofluorescence staining of the clones for CD117 showed that clonal cells continued to express CD117 (Fig. 3b). Furthermore, flow cytometer analysis confirmed that ~ 97% of the clonal cells expressed CD117 (Fig. 3c), suggesting CD117^+^ TSCs have the capacity of self-renewal in vitro*.* Finally, the expression of TSC marker genes [44] was assessed in total RNA extracted from CD117^+^ cells. Additional file 5: Figure S5 demonstrates by qRT-PCR that CDX2, EOMES, and ESRRB were all expressed in the cells. The levels of CDX2 and EOMES were significantly increased compared with MSCs (a stromal/stem cell also found in the placenta), while the expression of ESRRB showed a trend for an increase, although no significant difference. Immunocytochemical staining for CDX2 protein was also performed (Fig. 3d), which demonstrated 97 ± 3% of CD117^+^ cells in culture stained positive for CDX2. These data support the characterization of CD117^+^ placental cells as TSCs.

### Differentiation of CD117^+^ TSCs into lung alveolar epithelial cells in vitro

**Fig. 4:**
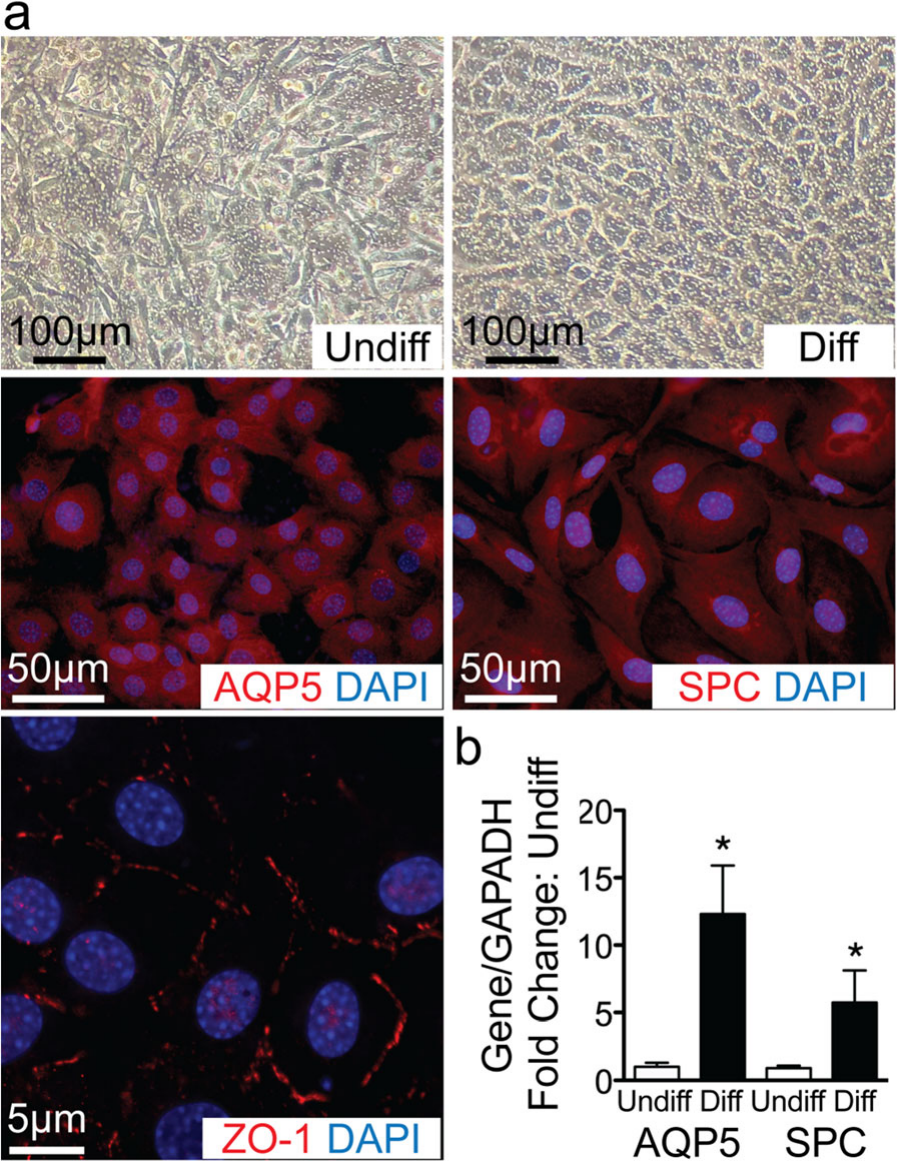
Differentiation of CD117^+^ TSCs into alveolar epithelial cells of lung in vitro. **a** Phase-contrast images showing morphologic change of cells before (Undiff, top row, left) and after differentiation (Diff, top row, right) in epithelial cell differentiation medium on air-liquid interface for 14 days. These differentiated cells were immunostained for AQP5 (red, middle row, left), SPC (red, middle row, right), and ZO-1 (red, bottom row, left). **b** Bar graph showing the quantitation of qRT-PCR for AQP5 and SPC in fold change comparing differentiated (Diff, black bars) to undifferentiated cells (Undiff, open bar). *P < 0.05 compared to their undifferentiated cells, *n* = 5 for each group

The placenta is an extra-embryonic organ that does not commit to a specific germ layer lineage. The capability of TCs to differentiate into tissue-resident cells originating from different germ cell layers is yet to be elucidated. Lung alveolar epithelial cells are derived from the embryonic endoderm. To determine whether CD117^+^TSCs have the potential to differentiate into cells of endodermal origin, we cultured these cells in differentiation medium of lung alveolar epithelial cells, on a transwell dish in an air-liquid interphase for 14 days. The morphology of the cells changed from a spider-like appearance (Fig. 4a top left, undifferentiated cells) into cobblestone-shaped cells (Fig. 4a, top right, differentiated cells). Immunofluorescence staining of the differentiated cells showed expression of AQP5 and SPC (Fig. 4a, middle row, left and right, respectively), markers for lung alveolar type I and II epithelial cells, respectively. Tight junctions were formed between these cells, as shown by staining for zonula occludens-1 (Fig. 4a, bottom, row left). Additionally, qRT-PCR revealed ~ 12-fold and ~ 5-fold greater mRNA levels of AQP5 and SPC respectively in the differentiated versus the undifferentiated cells (*P* < 0.05, Fig. 4b, respectively).

### Differentiation of CD117^+^ TSCs into cardiomyocytes in vitro

**Fig. 5:**
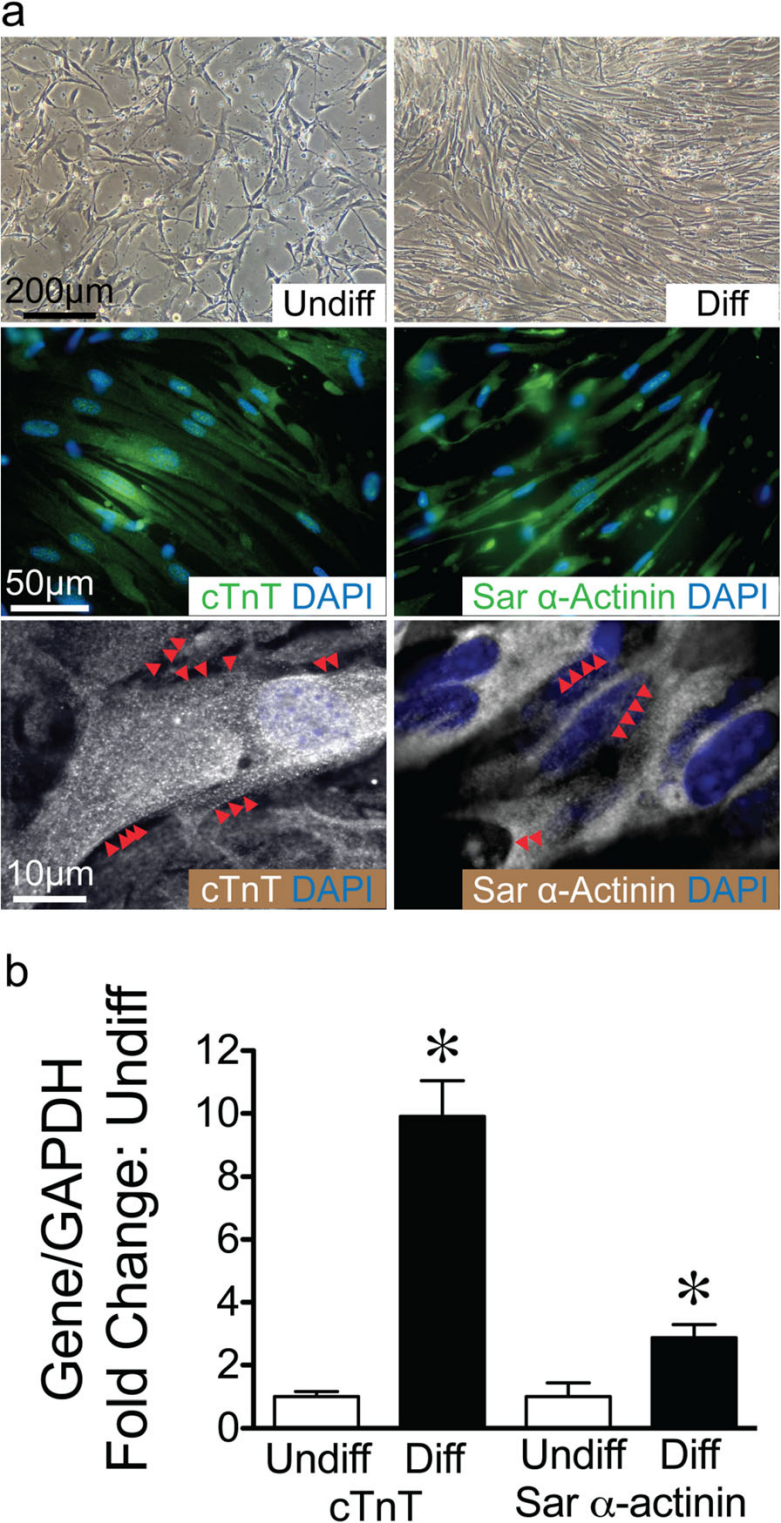
Differentiation of CD117^+^ TSCs into cardiomyocytes in vitro*.***a** Phase-contrast images showing morphologic change of cells before (Undiff, top row, left) and after differentiation (Diff, top row, right) in cardiomyocyte differentiation medium for 12 days. After differentiation, representative images of immunofluorescence staining for cTnT (green, middle row, left) and Sar α-actinin (green, middle row, right), merged with DAPI for nuclear staining (blue). Bottom panels show the confocal images of differentiated cells staining for cTnT (white, bottom row, left) and Sar α-actinin (white, bottom row, right). Red arrows highlight areas of striations. **b** Bar graph showing the quantitation of qRT-PCR for cTnT and Sar α-actinin in fold change comparing differentiated (Diff, black bars) to undifferentiated cells (Undiff, open bar). * *P* < 0.05 compared to their undifferentiated cells, *n* = 4~5 for each group

Cardiomyocytes are derived from the embryonic mesoderm. To investigate the potential of CD117^+^ TSCs to differentiate into mesoderm, we cultured the cells in cardiomyocyte differentiation medium for 7 days. Cells morphologically changed from a spider-like to a myofiber-like appearance in the differentiation medium (Fig. 5a top left, right, respectively). Differentiated cells stained positive by immunofluorescence for cTnT (Fig. 5a, middle row, left) and sarcomeric α-actinin (Fig. 5a, middle row, right), markers of cardiomyocytes. CD117^+^ TSCs were also differentiated in conditioned medium of neonatal heart cells for 3 weeks, and confocal microscopy was performed to show evidence of striations in the cells after cardiomyocyte differentiation, when stained for cTnT and sarcomeric α-actinin (Fig. 5a, bottom row, left and right panels respectively). qRT-PCR of total RNA extracts demonstrated that the levels of cTnT and sarcomeric α-actinin increased ~ 10-fold and ~ 3-fold respectively in differentiated cells, compared with undifferentiated cells (Fig. 5b).

### Differentiation of CD117^+^ TSCs into neuron photoreceptor cells in vitro

**Fig. 6:**
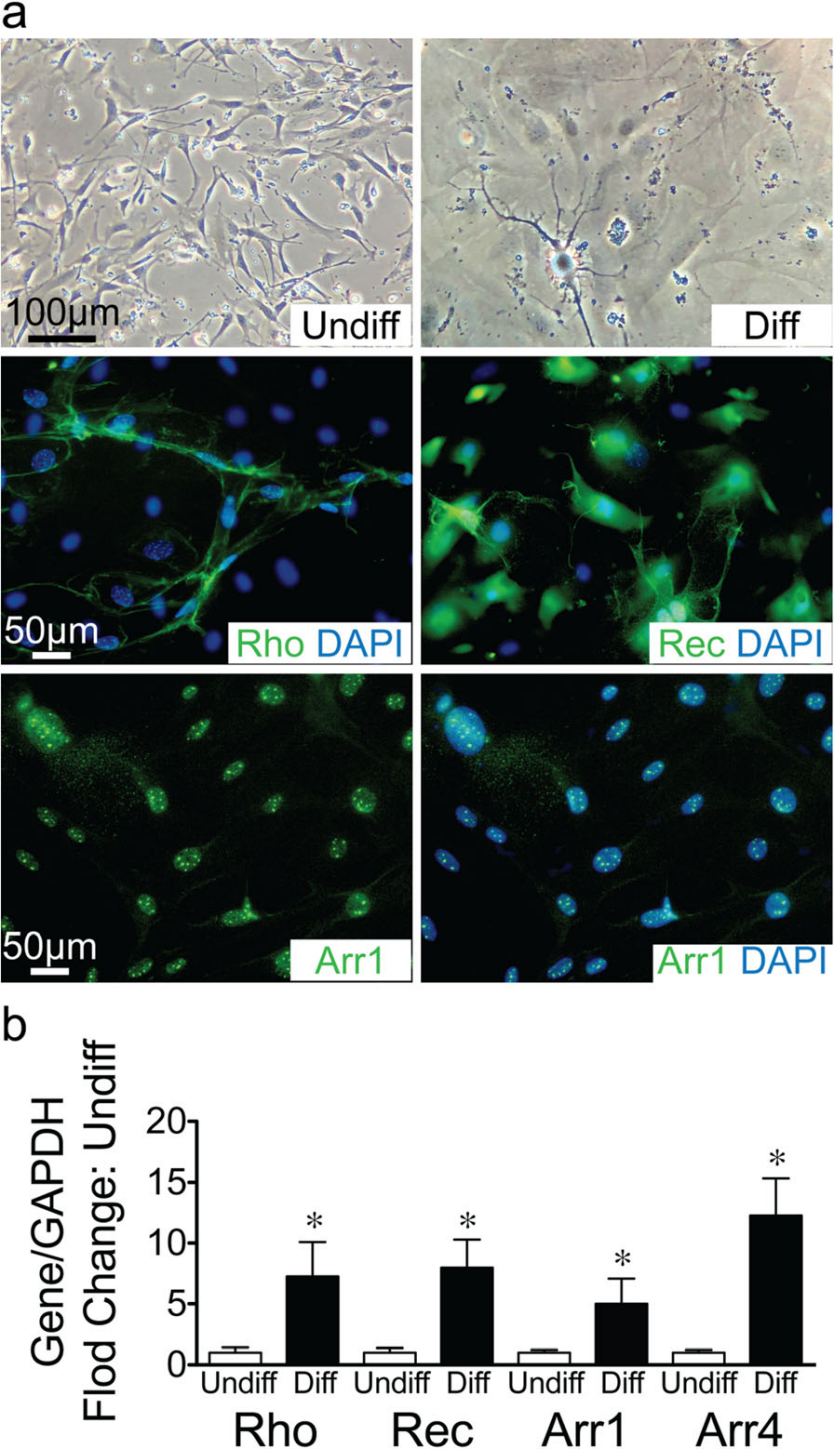
Differentiation of CD117^+^ TSCs into retinal photoreceptor cells in vitro. **a** Phase-contrast images showing morphologic change of cells before (Undiff, top row, left) and after differentiation (Diff, top row, right) in neuronal differentiation medium for 8 days. After differentiation, representative image of immunofluorescence staining of differentiated cells for Rho (green, middle row, left), Rec (green, middle row, right), and merged with DAPI for nuclear staining (blue). Arrestin 1 (Arr1, green, bottom row, left) and merged with DAPI for nuclear staining (blue, bottom row, right). **b** Bar graph showing quantitation of qRT-PCR for Rho, Rec, Arr 1 and Arrestin 4 (Arr4) comparing fold change in differentiated (Diff, black bars) versus undifferentiated cells (Undiff, open bar). *P < 0.05 compared to their undifferentiated cells, *n* = 4 for each group

The retina is the part of the central nervous system and derived from the embryonic ectoderm. CD117^+^ TSCs were cultured in a medium to induce retinal photoreceptor cell differentiation for 8 days. Cells morphologically changed from the spider-like (Fig. 6a top left) into a dendritic-like appearance in the differentiated cells (Fig. 6a top right). Immunofluorescence staining found that differentiated cells stained positive for rhodopsin and recoverin (Fig. 6a, middle row), and arrestin1 (Fig. 6a, bottom row), all markers of retinal photoreceptor cells. qRT-PCR also revealed significant increases in the levels of rhodopsin, recoverin, arrestin 1, and arrestin 4 (also a marker of retinal photoreceptor cells), in differentiated versus undifferentiated cells (Fig. 6b).

### Differentiation of CD117^+^ TSCs into three germ layers in vitro using the spheroid formation assay

**Fig. 7:**
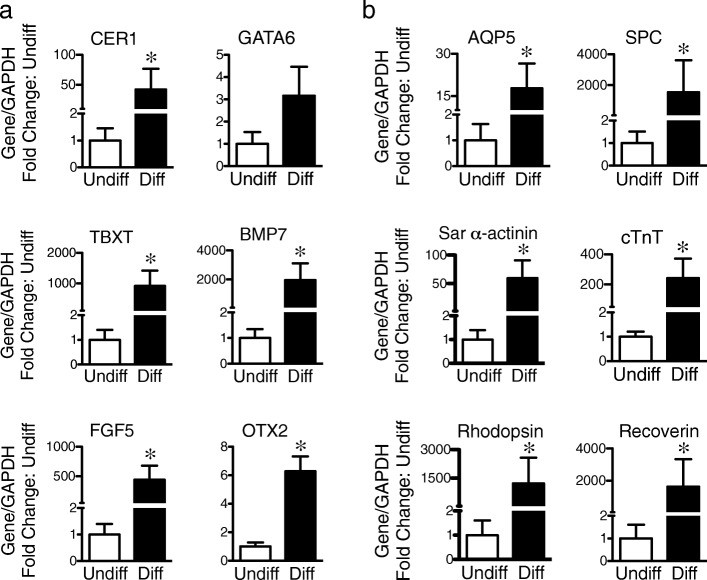
Differentiation of CD117^+^ TSCs into three germ layers in vitro. CD117^+^ TSCs were cultured by a hanging drop technique for 2 days and then the spheroids were cultured in ultra-low attachment 6-well plates for 14 days in growth medium in the absence of LIF and FGFβ. Total RNA was then extracted from the cells. **a** Bar graph showing quantitation of qRT-PCR for fold change in markers of endoderm (CER1 and GATA6), mesoderm (TBXT and BMP7), and ectoderm (FGF5 and OTX2). **b** Bar graph showing quantitation of qRT-PCR for fold change in markers of endoderm-derived lung alveoli epithelium cells (AQP5 and SPC), mesoderm-derived cardiomyocytes cells (Sar α-actinin and cTnT), and ectoderm-derived retinal photoreceptor cells (Rho and Rec). *P < 0.05 compared to their undifferentiated cells, *n* = 4 for each group

To determine whether the CD117^+^ TSCs had a differentiation bias for a specific germ layer, TSCs were induced to spontaneously differentiate by spheroid formation in culture in the absence of LIF, FGFβ, and other lineage-specific stimuli. RNA was extracted from the spheroids, and qRT-PCR performed for markers of endoderm (GATA6, CER1), mesoderm (TBXT, BMP7), and ectoderm (FGF5, OTX2). In comparison with undifferentiated CD117^+^ TSCs, the cells after spheroid formation demonstrated increased expression of markers for all three lineages—endoderm, mesoderm, and ectoderm (Fig. 7a). Moreover, after spheroid formation as compared with undifferentiated CD117^+^ TSCs, we found increased expression of SPC and AQP5 (endoderm-derived lung epithelial cells), cTnT and sarcomeric α-actinin (mesoderm-derived cardiomyocytes), and rhodopsin and recoverin (ectoderm-derived photoreceptors) (Fig. 7b). Taken together, these data suggest no strong bias for differentiation of CD117^+^ TSCs into a specific germ layer.

### Engraftment and differentiation of CD117^+^ TSCs into cells of three germ layers in vivo by in utero injection of cells

To exclude a non-specific finding of dye leaking into surrounding cells, we dyed the CD117^+^ TSCs with PKH67 (green) and then cocultured these cells with primary neonatal cardiac progenitor cells (CPCs) stained with a fluorescently labeled antibody to Sca-1 (red). We have previously demonstrated that > 90% of CPCs express Sca-1 [37]. The TSCs (green) and CPCs (red) were then cocultured for 5 h (1 TSC for every 10 CPCs). After the incubation period, the cells were detached from the plate into a suspension, and a cytospin was performed (to concentrate the cells). Imaging was then performed using a fluorescent microscope to determine whether any of the Sca-1 positive CPCs (red) took up PKH67 dye (green). Additional file 6: Figure S6 shows that there was no overlap of red and green fluorescent staining in any of the cells. These data demonstrated that TSCs dyed with PKH67 do not result in green staining of the surrounding cells within the first few hours.

**Fig. 8:**
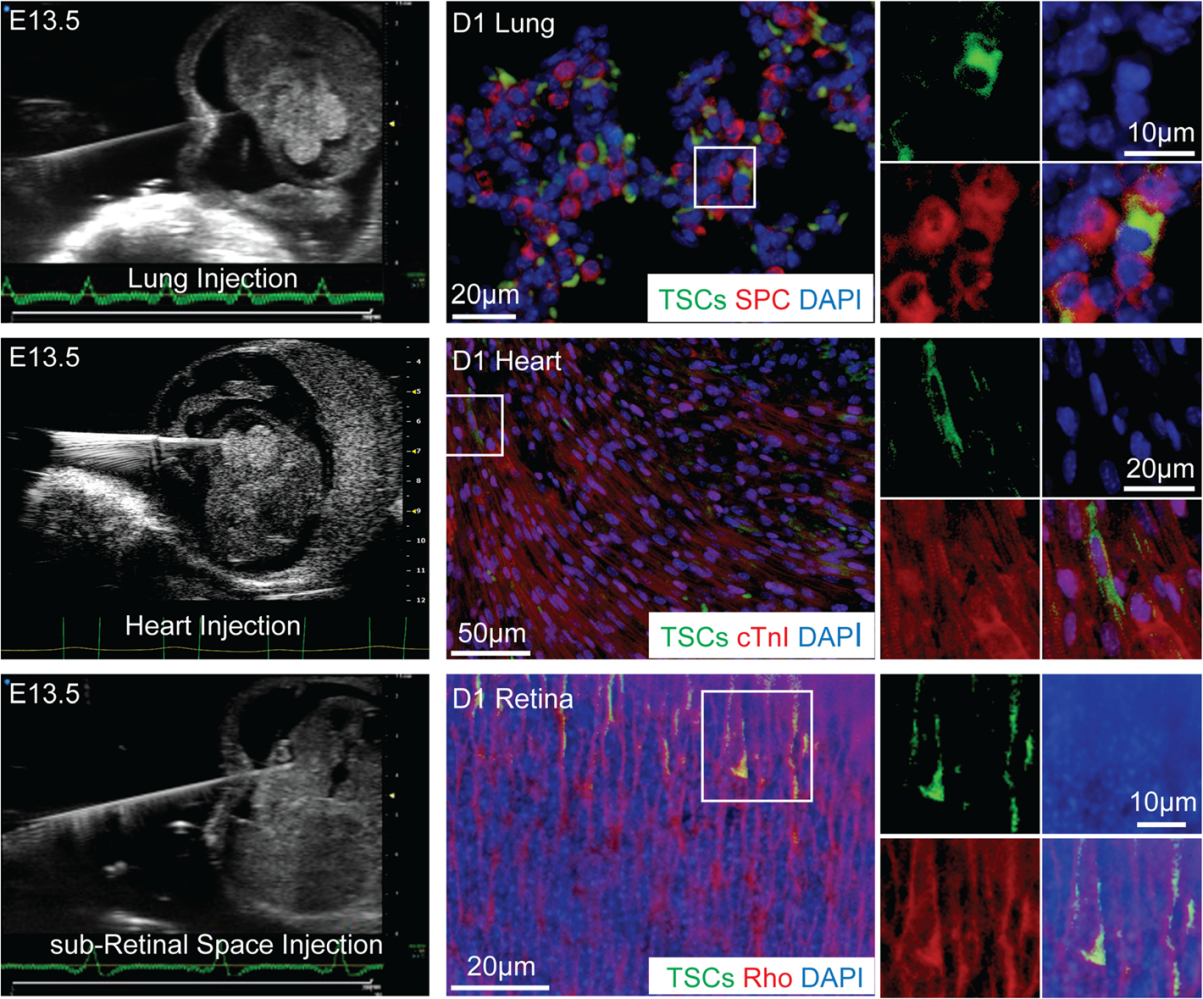
Differentiation of CD117^+^ TSCs into three germ layers in vivo**.** Under ultrasound guidance (left column), CD117^+^ TSCs pre-labeled with PKH67 (green) were injected in utero into lung, myocardium, or the sub-retinal space of E13.5 mouse fetuses. Lungs, hearts, and eyes were harvested at day 1 after nature birth for histological assessment. Representative merged images of exogenous cells (TSCs, green), DAPI (blue), and immunofluorescence staining for a marker of lung alveolar epithelium, SPC (red, upper row), or a marker of cardiomyocytes, cTnI (red, middle row), or a marker of retinal photoreceptors, Rho (red, lower row). The third and fourth columns show respectively higher power magnification images of the area in the white boxes of the second column

With the use of ultrasound guidance, transplantation of stem cells in utero is feasible [37, 45]. Our previous study demonstrated that in utero transplantation of CPCs into the fetal heart led to a high level of engraftment and differentiation of the progenitor cells into mature cardiomyocytes [37]. These data suggested that the fetal environment allows a favorable environment to promote engraftment and differentiation of exogenous progenitor cells. Thus, in the present study, we investigated the multipotent capacity of CD117^+^ TSCs in utero. CD117^+^ TSCs were pre-labeled with PKH67 (green fluorescence) and injected into lungs, hearts, and the sub-retinal space of E13.5 mouse fetuses under ultrasound guidance (Fig. 8, the first column, respectively). The three organs were harvested from day 1 pups after a natural delivery (6 days post-injection). Immunofluorescence staining of the organs was performed for assessment of CD117^+^ TSC engraftment and differentiation. Exogenous cells (green) were found to engraft into lung tissue and express SPC (Fig. 8, top row). The heart sections demonstrated immunofluorescence staining for cTnI in the green exogenous cells (Fig. 8, middle row). Lastly, we assessed neonatal retinas and found that green cells injected into the sub-retinal space migrated into the outer layer of the retina and expressed rhodopsin, a marker for photoreceptors (Fig. 8, bottom row). Taken together, these data demonstrate the capability of CD117^+^ TSCs to engraft and differentiate into lung, heart, and retinal parenchymal cells in utero*.*

## Discussions

Over the last decade, there has been a growing interest in the placenta as an available source of stem/progenitor cells. The placenta is discarded tissue following the completion of pregnancy, thus there are none of the ethical concerns surrounding the use of other embryonic tissues, and placental-derived cells carry less risks for age- and environmental-related DNA damage, as well as less tumorigenesis [7, 9–11, 46]. An additional advantage of placental cells is the demonstrated property of immune privilege, allowing the use of allogeneic cells for therapeutic intervention [6–8, 47, 48].

When the fertilized egg is implanted into the maternal uterus, the outer layer of the blastocyst becomes TCs consisting of two layers of cells; the inner cytotrophoblast cells and the outer syncytiotrophoblast cells that differentiate from the TSCs. The TSCs continue to proliferate and differentiate during the growth and development of the placenta to form other types of TCs, suggesting that TSCs have the property of stem cells [49–54]. TSCs are a heterogeneous population of cells with both differentiated and undifferentiated phenotypes [55]. Natale et al. found that undifferentiated trophoblasts, from mid-gestation mouse placentas, expressed the murine cell surface protein Sca1. The Sca1^+^ subpopulation of TCs demonstrated proliferation and multipotency with differentiation into TC types of both the junctional zone and the labyrinth layer [55]. In an attempt to identify a homogeneous subpopulation of undifferentiated TSCs in the near-term placenta with the potential application for isolation of these cells from mouse, and human placentas in the future, we assessed TSCs for the expression of CD117, a cell surface marker of stem/progenitor cells [30, 31]. Immunofluorescence staining demonstrated that a considerable number of cells in the labyrinth zone and chorionic plate expressed CD117. Additionally, immunofluorescence staining and flow cytometric analysis revealed a high percentage of CD117^+^ TSCs also expressed Sca-1 (> 70%), but did not express markers of hematopoietic cells (Figs. 1c and 2a, b) or MSCs (Figs. 1d and 2a, b). Interestingly, of the Sca1^+^ cells, only a portion express CD117 (Additional file 4: Figure S4). Thus, this trophoblast population of CD117^+^ cells represents a subpopulation of Sca1^+^ cells in the labyrinth zone and chorionic plate of the mouse placenta. The unique advantage of CD117 is that this stem cell marker is expressed in both mouse and human cells.

CD117^+^ cells have previously been found in mouse and human placentas [12, 18, 32–34] and isolated from AECs of human term placentas. AECs showed multipotent potential of endodermal differentiation into alveolar epithelium, pancreatic and hepatic cells, and mesodermal differentiation into osteocytes and chondrocytes [56–58]. However, the capacity of CD117^+^ TSCs to proliferate, and their differentiation potential outside the placenta remained unknown. When CD117^+^ TSCs were cultured at the limited dilution in the growth medium in vitro, the efficiency of colony formation was much higher in CD117^+^ TSCs (21.33 ± 3.09%, Fig. 3a) than that of adult stem cells [36, 37]. In addition, ~ 97.4% of the clonal cells continued to express CD117 (Fig. 3b, c), implicating the self-renewal property of CD117^+^ TSCs without initiating lineage differentiation, an important property of cells for use in therapy, when attempting to regenerate more than one lineage of cells.

Compared to the pluripotency of embryonic stem cells that possess the capacity to differentiate into all three germ layers, adult stem cells are more limited in differentiation potential. While the placenta is an extra-embryonic organ, we wanted to determine whether CD117^+^ TSCs were also capable of differentiating into cells of all three germ layers. Our experiments in vitro demonstrated that the CD117^+^ TSCs were able to differentiate into lung alveolar epithelial cells (endoderm, Fig. 4), cardiomyocytes (mesoderm, Fig. 5), and retinal photoreceptor cells (ectoderm, Fig. 6). To further understand the clinical application of CD117^+^ TSCs, we transplanted the cells into the lungs, hearts, and eye sub-retinal spaces of mouse fetuses at E13.5, under ultrasound guidance (Fig. 8). Immunofluorescence staining demonstrated that CD117^+^ TSCs were able to engraft into the lungs, hearts, and retinas of mouse fetuses, and they differentiated into alveolar epithelial cells, cardiomyocytes, and retinal photoreceptors, corresponding with the organs in which they were injected, by day 1 after birth. These data demonstrated that CD117^+^ TSCs have the capacity of differentiating into cells of all three germ layers in vitro and in vivo*.*

The present study advances our understanding of TSCs, especially as it applies to their ability to differentiate into parenchymal cells of organs outside of the placenta, both in vitro and in vivo. However, the present studies are limited in that they were not performed in a model of disease, thus the cells were not administered under injurious conditions. Further studies will need to be performed to understand the effects of TSCs under injurious conditions, and whether the actions of TSCs reflect their engraftment and differentiation potential, or whether paracrine actions contribute to a TSC response. While it has been suggested that placenta-derived cells are less likely to form tumors than ESCs and iPSCs [7, 9–11, 46], longer-term follow-up studies for safety after transplantation in mice will need to be performed.

## Conclusions

Taken together, our findings reveal a CD117^+^ subpopulation of mouse placenta-derived TSCs that have stem cell properties. These cells are self-renewing, clonogenic, and multipotent with the ability to differentiate into cells of three germ layers in vitro and in vivo. The CD117 surface marker provides a straightforward means to isolate this subpopulation of undifferentiated cells. In utero transplantation of CD117^+^ TSCs into the lungs, hearts, and eye sub-retinal spaces of mouse fetuses resulted in cell engraftment, and differentiation into organ-specific parenchymal cells by the day of birth. We propose that the ability to specifically isolate a homogeneous population of TSCs, using CD117 as a marker, and expanding these cells in vitro, will provide an allogeneic source of stem cells for therapeutic strategies in the future.

## Supplementary information

Additional file 1: **Figure S1.** Dividing cells in the labyrinth zone and chorionic plate of the mouse placenta. Embryonic day 18.5 placenta immunofluorescence staining for Ki67 (red), a marker for dividing cells. The right panels demonstrate merged images of Ki67 and DAPI (blue) in the labyrinth (top row) and chorionic plate (bottom row).

Additional file 2: **Figure S2.** Growth Curve of CD117^+^ trophoblast stem cells. 15,000 TSCs were plated on a 35-mm2 dish (triplicate), and cells were harvested for counting every day for 5 days. Fold increase was calculated compared with day 0 (15,000 cells). n=5.

Additional file 3: **Figure S3.** Expression of Sca1 in CD117^+^ cells. Cells were isolated from embryonic day 18.5 placentas using CD117-immnune microbeads, and then expanded in growth medium in a culture dish. The cells were lifted from the dish, resuspended, and stained using CD117 antibody (Abcam, Cat. ab64677, 1:30) and then incubated with species-matched secondary antibodies conjugated with Tetramethylrhodamine (TRITC, Jackson Immuno Res, Cat. 711-025-152, 1:50). After CD117 staining, cells were stained using Sca1 conjugated with fluorescein isothiocyanate (FITC, eBioscience, Cat. 11-5981, 1:100). DAPI was added to stain nuclei following fixation of the cells, and then a cytospin of the cells was performed onto a slide. Images were taken by fluorescence microscopy. Representative images showed the cells were positive for CD117 (red, top and bottom panels) and Sca1 (green, middle and bottom panels). The bottom panels demonstrated merged images of CD117, Sca1 and DAPI (blue). Right column showed in higher power images from the area of white boxes in left columns.

Additional file 4: **Figure S4.** Expression of CD117 and Sca1 in trophoblast cells. Trophoblast cells (TCs) were isolated from embryonic day 18.5 placentas using a percoll gradient, and expanded in growth medium. Sca1 antibody conjugated with fluorescein isothiocyanate (FITC, eBioscience, Cat. 11-5981, 1:100) and CD117 antibody conjugated with allophycocyanin (APC, BD Pharmingen, Cat. 553356, 1:10) were incubated with the TCs at 4ºC for 30 min in darkness. DAPI was added to stain nuclei following fixation of the cells, and then a cytospin of the cells was performed onto a slide. Images were taken by confocal microscopy, with a lower power image on the top row, and cells within the white boxes depicted in a higher power image on the lower row. Representative images showed that the majority of TCs were positive for Sca1 (green, left and right columns). A subpopulation of Sca1+ cells also expressed CD117 (red, middle and right columns). Right column showed merged images of Sca1, CD117 and DAPI (blue).

Additional file 5: **Figure S5.** Gene expression of CD117^+^ trophoblast stem cells (TSCs). Total RNA was extracted from mouse mesenchymal stromal cells (MSC, white bar) and CD117^+^ TSC (black bar). Quantitative polymerase chain reaction was performed and gene expression was normalized by GAPDH. Fold change was compared to MSC. * P<0.05 TSC versus MSC.

Additional file 6: **Figure S6.** Assessment of PKH67 dye leakage into surrounding cells in vitro. CD117+ TSCs were dyed with PKH67 (green, left upper panel) and cardiac progenitor cells (CPCs) were incubated with anti-Sca1 antibody conjugated with Alex 555 (red, left lower panel). TSCs (green) were mixed with CPCs (red) at a ratio of 1:10 and co-cultured for 5 hours. Cells were harvested and a cytospin performed to concentrate the cells. Representative image showing there is no overlap of green and red fluorescent staining in any of the cells. Merged image of green, red, and blue (DAPI staining for nuclei) shown in right panel. White arrows highlight the green TSCs.

## Data Availability

The original data are available from the corresponding author on request.

## Abbreviations

AECs: Amniotic epithelial cells
AQP5: Aquaporin 5
Arr1: Arrestin 1
Arr4: Arrestin 4
BMP7: Bone morphogenetic protein 7
CDX2: Caudal type homeobox 2
CER1: Cerberus
CK7: Cytokeratin 7
cTnI: Cardiac troponin I
cTnT: Cardiac troponin T
DAPI: 4′,6-Diamidino-2-phenylindole
ECAM: E-cadherin
EOMES: Eomesodermin
ESRRB: Estrogen-related receptor beta
FGF: Fibroblast growth factor
GATA6: GATA-binding protein 6
HSCs: Hematopoietic stem cells
MHC: Classical major histocompatibility complex
MSCs: Mesenchymal stromal cells
OTX2: Orthodenticle homeobox 2
qRT-PCR: Real-time polymerase chain reaction
Rec: Recoverin
Rho: Rhodopsin
Sar α-actinin: Sarcomere α-actinin
SPC: Prosurfactant protein C
TBXT: T-box transcription factor T
TCs: Trophoblast cells
TSCs: Trophoblast stem cells
VMT: Vimentin
ZO-1: Tight junction protein-1
