# Supplementary figures and images for: Multipotency of mouse trophoblast stem cells
Source: Stem Cell Res Ther. 2020 Feb 13;11:55. doi: 10.1186/s13287-020-1567-4 (PMC7020558; doi:10.1186/s13287-020-1567-4)

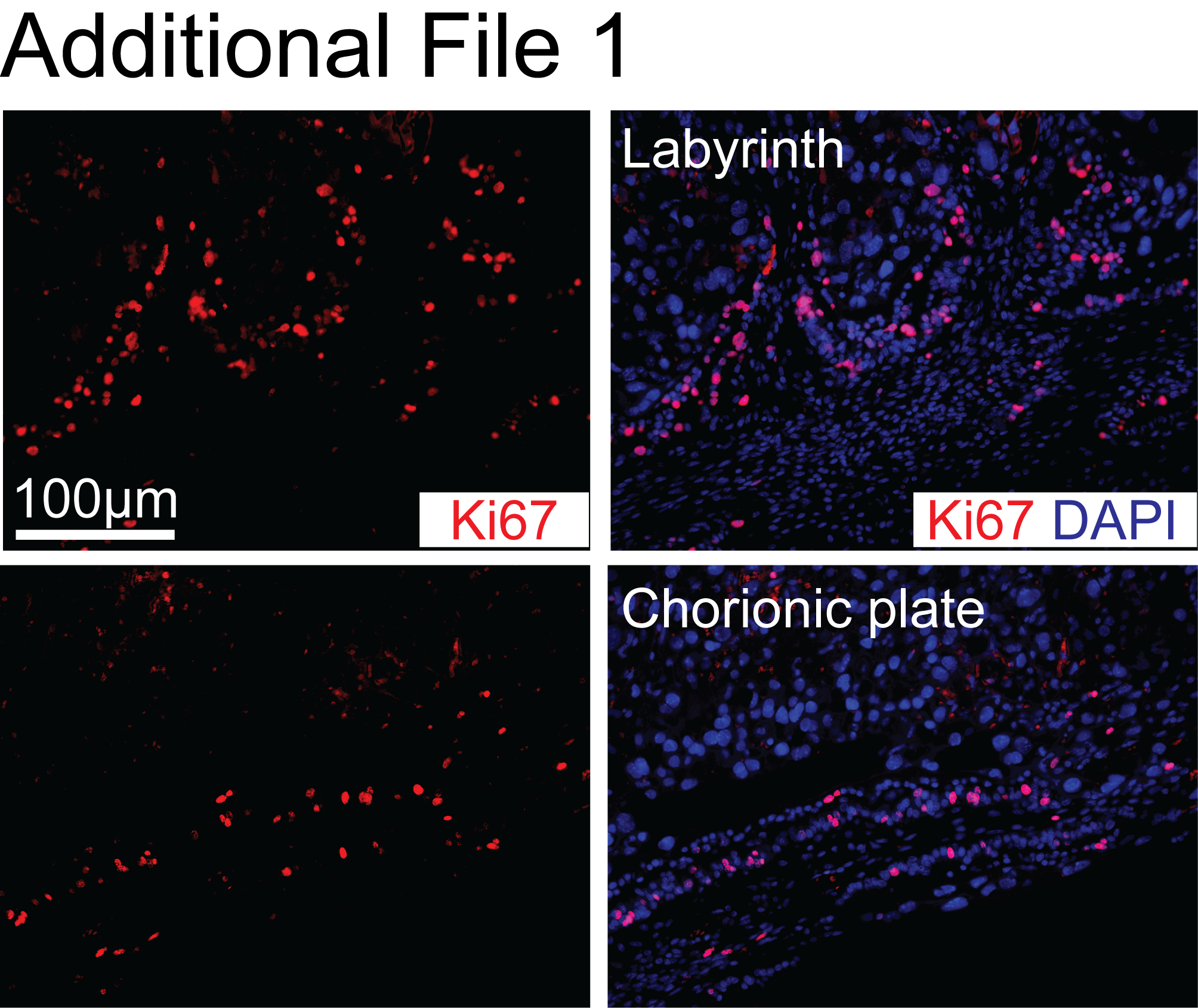

Supplement: Supplementary file 1 — Figure S1. Dividing cells in the labyrinth zone and chorionic plate of the mouse placenta. Embryonic day 18.5 placenta immunofluorescence staining for Ki67 (red), a marker for dividing cells. The right panels demonstrate merged images of Ki67 and DAPI (blue) in the labyrinth (top row) and chorionic plate (bottom row). [file 13287_2020_1567_MOESM1_ESM.png]

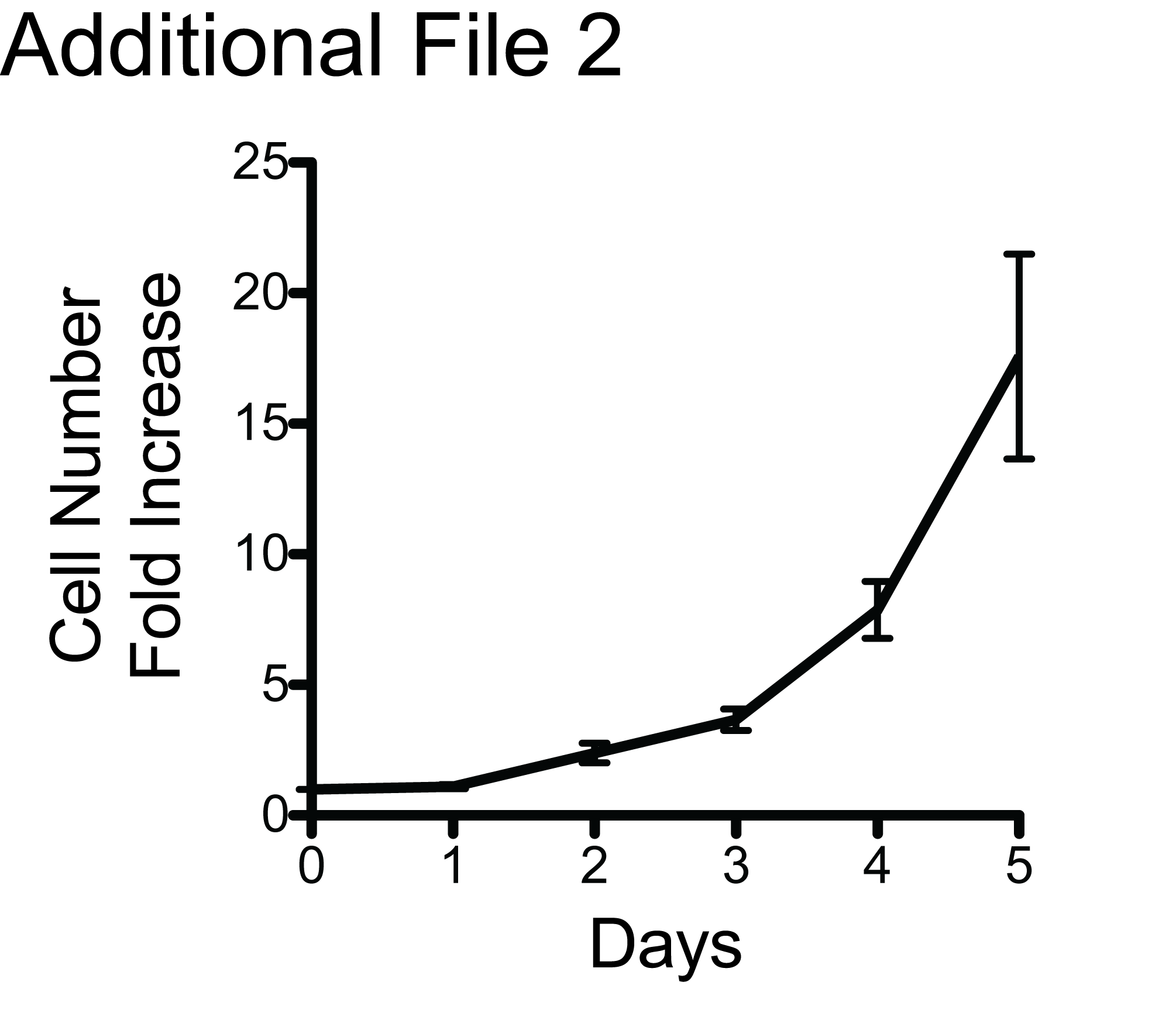

Supplement: Supplementary file 2 — Figure S2. Growth Curve of CD117+ trophoblast stem cells. 15,000 TSCs were plated on a 35-mm2 dish (triplicate), and cells were harvested for counting every day for 5 days. Fold increase was calculated compared with day 0 (15,000 cells). n=5. [file 13287_2020_1567_MOESM2_ESM.png]

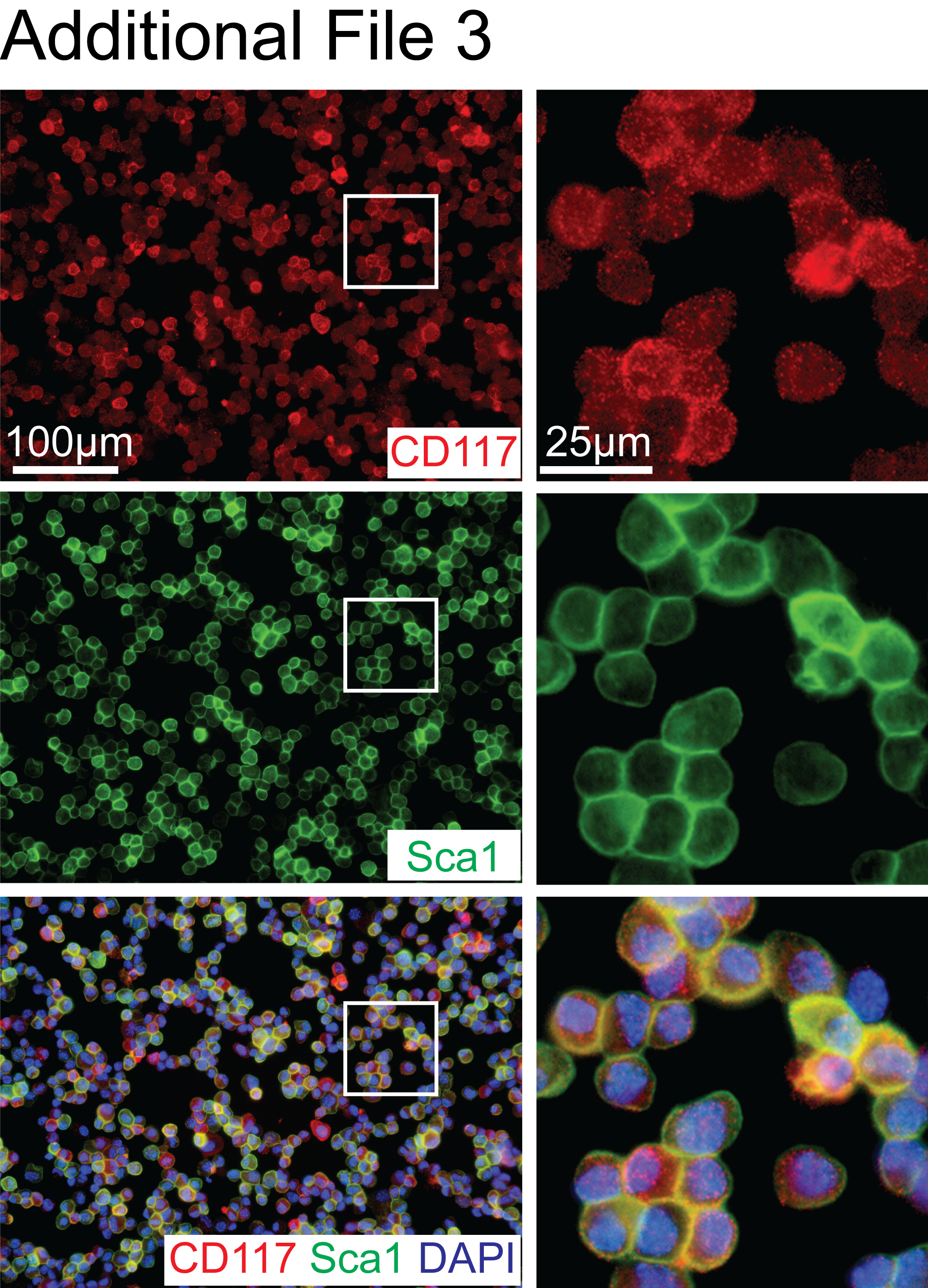

Supplement: Supplementary file 3 — Figure S3. Expression of Sca1 in CD117+ cells. Cells were isolated from embryonic day 18.5 placentas using CD117-immnune microbeads, and then expanded in growth medium in a culture dish. The cells were lifted from the dish, resuspended, and stained using CD117 antibody (Abcam, Cat. ab64677, 1:30) and then incubated with species-matched secondary antibodies conjugated with Tetramethylrhodamine (TRITC, Jackson Immuno Res, Cat. 711-025-152, 1:50). After CD117 staining, cells were stained using Sca1 conjugated with fluorescein isothiocyanate (FITC, eBioscience, Cat. 11-5981, 1:100). DAPI was added to stain nuclei following fixation of the cells, and then a cytospin of the cells was performed onto a slide. Images were taken by fluorescence microscopy. Representative images showed the cells were positive for CD117 (red, top and bottom panels) and Sca1 (green, middle and bottom panels). The bottom panels demonstrated merged images of CD117, Sca1 and DAPI (blue). Right column showed in higher power images from the area of white boxes in left columns. [file 13287_2020_1567_MOESM3_ESM.png]

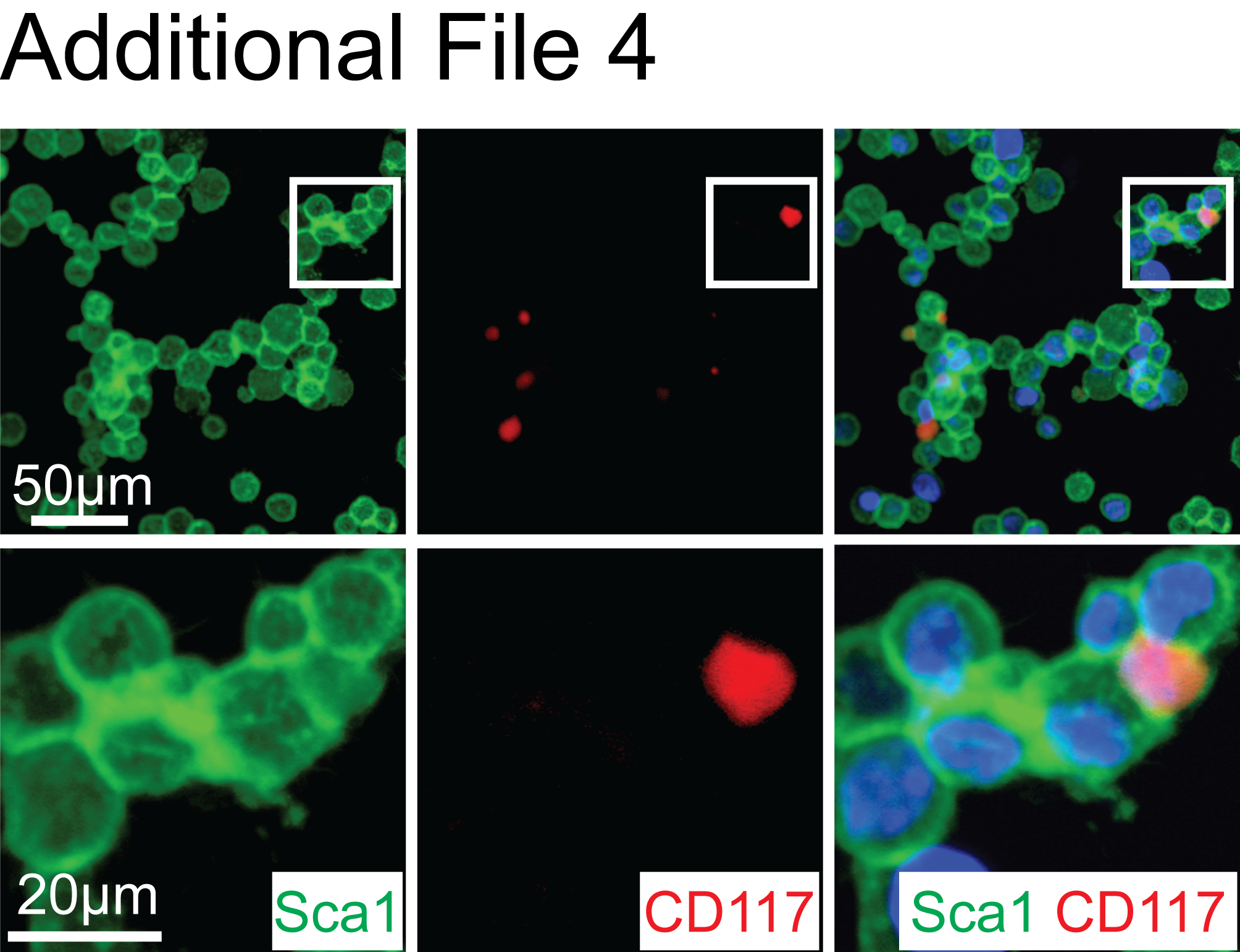

Supplement: Supplementary file 4 — Figure S4. Expression of CD117 and Sca1 in trophoblast cells. Trophoblast cells (TCs) were isolated from embryonic day 18.5 placentas using a percoll gradient, and expanded in growth medium. Sca1 antibody conjugated with fluorescein isothiocyanate (FITC, eBioscience, Cat. 11-5981, 1:100) and CD117 antibody conjugated with allophycocyanin (APC, BD Pharmingen, Cat. 553356, 1:10) were incubated with the TCs at 4ºC for 30 min in darkness. DAPI was added to stain nuclei following fixation of the cells, and then a cytospin of the cells was performed onto a slide. Images were taken by confocal microscopy, with a lower power image on the top row, and cells within the white boxes depicted in a higher power image on the lower row. Representative images showed that the majority of TCs were positive for Sca1 (green, left and right columns). A subpopulation of Sca1+ cells also expressed CD117 (red, middle and right columns). Right column showed merged images of Sca1, CD117 and DAPI (blue). [file 13287_2020_1567_MOESM4_ESM.png]

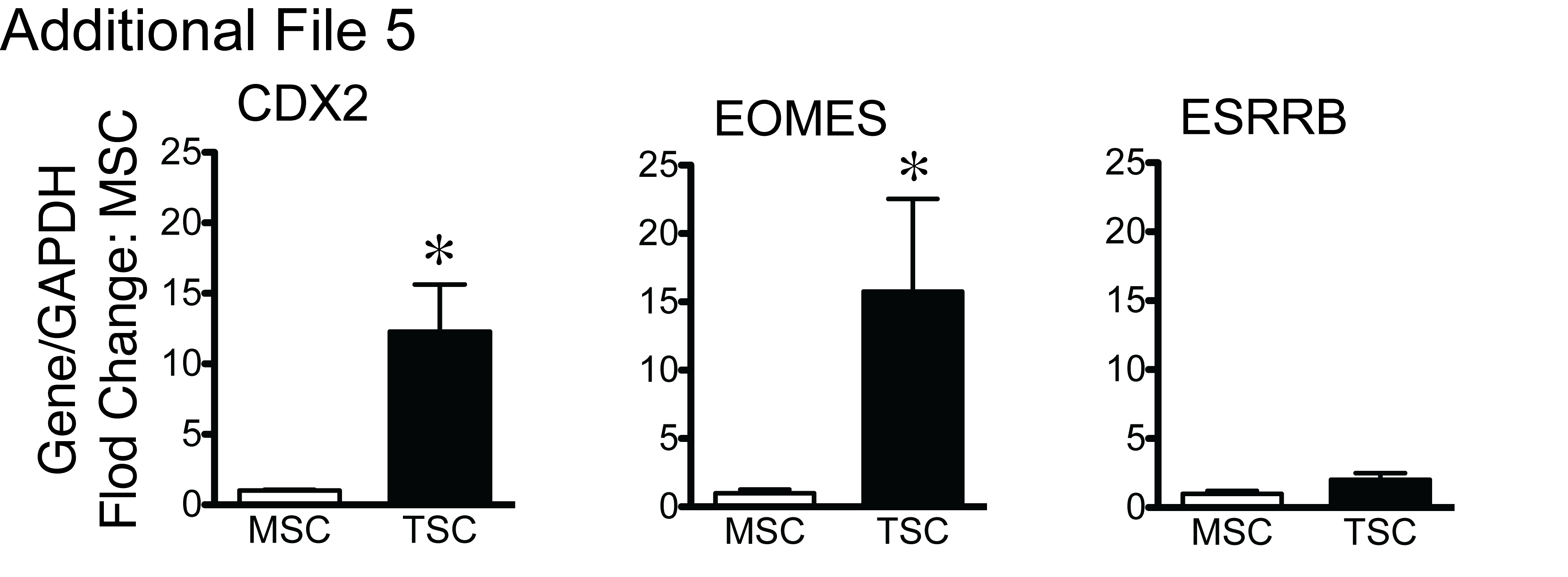

Supplement: Supplementary file 5 — Figure S5. Gene expression of CD117+ trophoblast stem cells (TSCs). Total RNA was extracted from mouse mesenchymal stromal cells (MSC, white bar) and CD117+ TSC (black bar). Quantitative polymerase chain reaction was performed and gene expression was normalized by GAPDH. Fold change was compared to MSC. * P<0.05 TSC versus MSC. [file 13287_2020_1567_MOESM5_ESM.png]

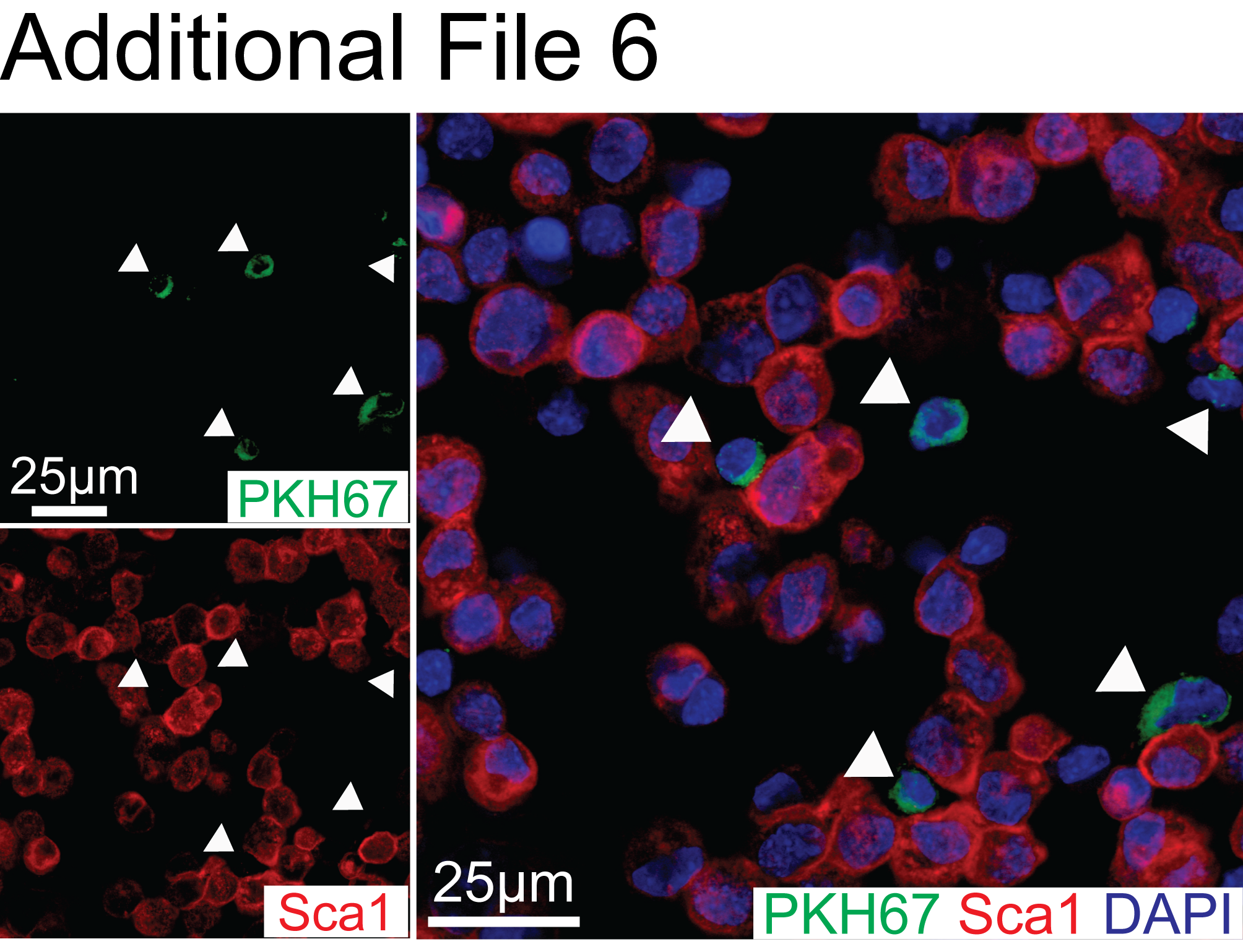

Supplement: Supplementary file 6 — Figure S6. Assessment of PKH67 dye leakage into surrounding cells in vitro. CD117+ TSCs were dyed with PKH67 (green, left upper panel) and cardiac progenitor cells (CPCs) were incubated with anti-Sca1 antibody conjugated with Alex 555 (red, left lower panel). TSCs (green) were mixed with CPCs (red) at a ratio of 1:10 and co-cultured for 5 hours. Cells were harvested and a cytospin performed to concentrate the cells. Representative image showing there is no overlap of green and red fluorescent staining in any of the cells. Merged image of green, red, and blue (DAPI staining for nuclei) shown in right panel. White arrows highlight the green TSCs. [file 13287_2020_1567_MOESM6_ESM.png]
